# The Clinical Congress of Surgeons of North America

**Published:** 1914-08-01

**Authors:** 


### THE PROFESSION OF MEDICINE.

### The Clinical Congress of Surgeons of North America.

The London institutional world has welcomed this week the invasion of some hundreds of visitors from across the Atlantic, who have come to attend the fifth Clinical Congress of Surgeons of North America. In the public mind there is probably some degree of confusion between this Congress and the International Medical Congress, which met in London about this time last year; but in reality the two are wholly distinct, not only in constitution, but in aims and methods. The object of the Clinical Congress of Surgeons, a young association in quite an early stage of growth, but remarkably vigorous for its age, is to study the art and science of surgery by visiting surgical clinics and watching as many different front-rank operators at work as possible. The Clinical Congress has no concern primarily with entertainments or amusements or dinners: it is out for business, and for essentially practical surgical sight-seeing all the time. Even the papers read at the evening sessions are of quite secondary importance, though these meetings are well attended, and the debates closely followed. The topics selected for them are carefully chosen for their practical character: it is common diseases or operations which are selected as texts for the discourses; and though original thought and work is naturally expected of those who read papers, it is not profoundly original research that is, as a rule, the keynote of the contributions, but rather a summing-up of present-day expert practice and approved surgical science.

### Some Impressions in the Clinics.

The best impression of our visitors is, as will be easily comprehended, to be obtained in the operating theatres of the clinics rather than in the big lecture-halls of the Cecil and Savoy Hotels. In thirty leading London hospitals, special and general, careful arrangements have been made for showing the visitors the actual state of London surgery; and it is in the theatres and ante-rooms that they are most at home. Speaking generally, the American (and Canadian) surgeons are well pleased with what they have seen; and this can be affirmed with the more confidence in that they do not hesitate to criticise anything which seems to them not quite up to the mark. Their expressions of approval may, in consequence, be taken at their face value, not as mere politeness and verbal courtesy; and from what the writer has seen and heard of their views, when they do offer criticisms there is always good ground for them. In other words, our surgeons are on trial before a jury of experts, whom no arts save those of honest, skilful surgery will sway, and whose verdict is impartial.

In thus emphasising the open-mindedness of the visitors, and their readiness to award either praise or blame as they may conceive right, no lack of courtesy must be imputed to the Congressers. Widely as they differ among themselves in age, nationality, appearance, dress, and speech, there is one characteristic common to them all—namely, their punctilious politeness and amiable address. No matter how tedious the minutiæ of some difficult and prolonged operation may be, not a movement nor a sound escapes the onlookers such as might embarrass the operator. The same patient forbearance has been noticeable at the evening meetings. One or two of the discussions have been wearisome to the last degree; but no sign of the boredom which the large audience must have felt on certain occasions was allowed to travel from the body of the hall to the platform. We fear that a collection of British surgeons "talked down to" as this Congress has once or twice been would scarcely have concealed so successfully its opinion of the speaker, or afforded him such generous applause on resuming his seat. There has been, and it is best to face it frankly—a tendency on the part of some of our surgeons, both in the clinics and in the Cecil Hotel, to treat the visitors on the level of junior medical students, and to lecture them as if they were wholly ignorant of the first principles of surgical technique. These airs of superiority have usually been displayed by men not quite in the very first rank of British surgery, which fact has naturally been detected by the vigilant watchers of their operative technique; it is foolish and hopeless to expect to deceive one's fellow-craftsmen by such exhibitions of littleness, and the attempts have, in fact, been complete failures.

Up to and including Wednesday the best of the formal papers read at the evening meetings has been that of Mr. Robert Jones, the Liverpool orthopædist, on "Internal Derangements of the Knee-Joint." The subject is painfully hackneyed in this country, but according to Mr. Jones has not been paid so much attention to in America. At all events he expounded it very carefully, thoroughly, and authoritatively in an address which was a model of correct composition and graceful delivery. In this latter respect there have been, as was only to be expected, wide individual divergences; and, broadly speaking, the British surgeons who have spoken have not displayed the same mastery over the arts of elocution and public speaking as the Continental and Transatlantic visitors have done. Professor Tuffier, for instance, whose English is not at all good, yet offered an example of dignified delivery, which extracted from the audience applause more prolonged than that which any other speaker received on Tuesday evening. It is a pity that so many British surgeons fail in this respect, and it might be suggested to those who make the arrangements for these functions that in choosing speakers regard should be paid to the reputation of the surgeon, not only for soundness of views, but also for the capacity to express them in logical sequence and in decent English. Possibly many of the visitors do not attach great importance to small points such as this, but nevertheless they should be attended to.

